# Event and Comment

**Published:** 1911-02-15

**Authors:** 


### DENTAL REGISTER.

**Vol. LXV. February 15, 1911. No. 2**

### EVENT AND COMMENT.

A JOURNAL OF ORAL HYGIENE. That the general subject of Oral Hygiene is making progress, we are encouraged to believe because a brand new dental journal has appeared to champion the cause. This journal is apparently put on an altruistic basis, since it is to be mailed to every dentist in the United States without cost to him. The publishers assuming all expense not only of editing and publishing, but of paying the postage as well. Since it can not be mailed at the usual magazine rates this is quite an item for forty thousand subscribers. The journal is to be edited by that versatile and brilliant author and editor Dr. George Edwin Hunt, of Indianapolis, who may not be known widely as an oral hygienist, but he has the faculty of grinding any grist that comes to his mill, and we are sure he will do this one in a unique and comprehensive way, if he doesn't "get tired and quit." In his salutatory he says, "you will receive *Oral Hygiene* twelve times a year, without money or price,—but I hasten to explain that this is in no sense due to the open-handed liberality and well-known benevolence of the Editor,—I am not paying the bills,—so don't thank me for it. It is all made possible by the publishers and advertisers. I have no idea how you can stop the magazine if you don't want it. Candidly I don't believe it can be done. You can always stop the ordinary Dental Journal by refusing to pay the price, but such a refusal would not affect us a bit, we don't care a whooper whether you want it or not. You had better accept it, as it may contain something some day that will be good for you. If you don't like it, let us know and we will change it to suit you. Anyhow it's coming to you."

Now some of our folks will probably get huffy and tell these editor and publisher people that we are not paupers; and perhaps some may also say that they are not interested in oral hygiene any way. But we don't see that this will help us out, we shall have to read George Edwin's compilations in spite of ourselves as it is bound to come every thirty days to the front door at least. We may stand back on our professional dignity, as the National Dental Association did last summer, and say that we will not allow the commercial house to put us under further obligations, even in behalf of so excellent a cause. But if we are to wait until the dental profession gives up money enough to do any great work, however benevolent and desirable, that costs much, we shall not soon become known as a benevolent profession. This is a grand movement, and if the dental profession can not or will not spread the propaganda in accordance with its traditional ideas of professionalism, it seems to us that the cause ought to be sufficiently ennobling to overshadow the methods of its propagandism. Since it is questionable if these are capable of being seriously criticised by those who care more for results than mere methods. We might hope that these things should be otherwise, perhaps, but since they arn't, lets wish the movement every success, and hope that it may be the means of arousing our profession to the greatness of this subject and the great need for its general co-operation to insure its success.

RESIGNATION FROM THE NATIONAL ASSOCIATION OF DENTAL EXAMINERS. We notice in the *Scrap Book* a statement that the New Jersey Board of Examiners has resigned its membership in the N. A. D. E. and gives the following reasons for so doing: First, it states that the National Association is no longer useful as an active body. Secondly, it has nullified its efficient Tabulating Committee by giving this work over to a joint committee composed of delegates from the National Dental Association and National Faculties Association and its own organization. Thirdly, the very efficient work done by Dr. Irwin of the N. J. Board has been discarded and will thus be lost to the profession. Lastly, the rules of the Association hinder and handicap the work of the N. J. Board. Now of course we don't know all the details of the affair and consequently can't referee the matter; but it seems to us that there should be some way of reconciling such slight objections as those given, and even if they could not be wholly adjusted concession might be made for a time until the present rules and regulations of the National Associations should have been tested. Perhaps the New Jersey men may then find the rules more wholesome than they anticipate. It seems to us that the National Association of Examiners is more needed just now than almost any other National organization, and that it ought to remain intact and have the benefit of the advice of the men who have been associated with the development of our present legal standards of registration. Our laws and the methods of enforcing them are not perfect as yet. There should be more uniformity and the establishment of comity in interstate relations for change of residence, and we should have a dignified standard of education for registration. All these desirable things could be brought to pass by this body more speedily than by any other. Every State Board in the country should join the National organization and make a concerted move for these desirable features in our registration laws. We trust the action of the New Jersey Board may be reconsidered, as this Board has always taken a most active part in the work of this organization and its advice is more needed now than ever before.

